# Mitochondrial-Targeting Anticancer Agent Conjugates and Nanocarrier Systems for Cancer Treatment

**DOI:** 10.3389/fphar.2018.00922

**Published:** 2018-08-17

**Authors:** Gantumur Battogtokh, Yong-Yeon Cho, Joo Young Lee, Hye Suk Lee, Han Chang Kang

**Affiliations:** Department of Pharmacy, College of Pharmacy, The Catholic University of Korea, Bucheon, South Korea

**Keywords:** mitochondria-targeting ligand-drug conjugate, mitochondria-targeted nanocarrier system, anticancer agents, triphenylphosphonium, doxorubicin, mitochondria

## Abstract

The mitochondrion is an important intracellular organelle for drug targeting due to its key roles and functions in cellular proliferation and death. In the last few decades, several studies have revealed mitochondrial functions, attracting the focus of many researchers to work in this field over nuclear targeting. Mitochondrial targeting was initiated in 1995 with a triphenylphosphonium-thiobutyl conjugate as an antioxidant agent. The major driving force for mitochondrial targeting in cancer cells is the higher mitochondrial membrane potential compared with that of the cytosol, which allows some molecules to selectively target mitochondria. In this review, we discuss mitochondria-targeting ligand-conjugated anticancer agents and their *in vitro* and *in vivo* behaviors. In addition, we describe a mitochondria-targeting nanocarrier system for anticancer drug delivery. As previously reported, several agents have been known to have mitochondrial targeting potential; however, they are not sufficient for direct application for cancer therapy. Thus, many studies have focused on direct conjugation of targeting ligands to therapeutic agents to improve their efficacy. There are many variables for optimal mitochondria-targeted agent development, such as choosing a correct targeting ligand and linker. However, using the nanocarrier system could solve some issues related to solubility and selectivity. Thus, this review focuses on mitochondria-targeting drug conjugates and mitochondria-targeted nanocarrier systems for anticancer agent delivery.

## Introduction

Mitochondria play important roles in living cells as an energy source and as a generator of reactive substrates, which are harmful to living cells. Therefore, targeting mitochondria is attracting great attention in medicine. The idea of mitochondrial targeting was posed in 1950 after determining the mitochondrial structure and some molecules with mitochondrial affinity (Palade, 1952, 1964; Ernster and Schatz, 1981). Delocalized lipophilic cations (DLCs), such as triphenylphosphonium, dequalinium, and rhodamine 123, were the first mitochondria-targeting molecules (Ernster and Schatz, 1981; Lampidis et al., 1985; Weiss et al., 1987; Christman et al., 1990; Dairkee and Hackett, 1991). Later, many studies used those ligands in mitochondria-targeted antioxidant and anticancer drug development. The concept of mitochondrial targeting is based on the mitochondrial membrane potential, which differs from the plasma membrane potential. The mitochondrial membrane potential is approximately -180 mV, which is 3- to 5-fold higher than that of the plasma membrane (Zhang and Zhang, 2016b; Zielonka et al., 2017). Thus, positively charged molecules easily accumulate in the mitochondria. However, since mitochondria have a four-layer structure (outer membrane, intermembrane space, inner membrane, and matrix), it is difficult for many molecules to enter the mitochondria. Therefore, lipophilic molecules are used due to their membrane-permeability, allowing the molecules to traverse the mitochondrial membranes by hydrophobic interactions. In several cases, the mitochondria of cancer cells and transformed cells exhibit significantly increased transmembrane potentials compared with normal cells (Dairkee and Hackett, 1991). This biological difference has been utilized as a basis to develop mitochondria-targeting compounds that may be preferentially accumulated within the mitochondria of cancer cells. An alternative option for cancer cell mitochondria targeting over normal cell mitochondria is its ability to generate high reactive oxygen species (ROS) and adenosine triphosphate (ATP) by aerobic glycolysis as compared to that in normal cells by Krebs cycle. Therefore, mitochondria are vulnerable to dysfunction and mitochondrial DNA mutation. Aerobic glycolysis in cancer cells is known as the Warburg effect (Wallace, 2005). Additionally, many anticancer drugs (doxorubicin, cisplatin, paclitaxel, resveratrol, and porphyrin derivatives) are known to be activated in the membrane and the matrix of mitochondria and by mitochondrial nucleases (Wang et al., 2010; Heller et al., 2012). If we can enhance the mitochondrial accumulation of anticancer drugs, the therapeutic effect could be improved, and drug resistance may be resolved. On the other hand, mitochondria targeting anticancer agents are called “mitocans,” which selectively accumulate in mitochondria and damage mitochondrial membrane, leading ROS generation and apoptotic cell death (Neuzil et al., 2013).

There are two well-known approaches for mitochondria drug delivery: direct conjugation of the targeting ligand to drugs and attachment of the targeting ligand to a nanocarrier. In this review, we discuss these approaches and recent achievements. In the case of direct conjugation of the targeting ligand to a drug, many aspects should be considered, including the type of linkage and the type of targeting ligand. There are a number of mitochondria-targeting conjugates, most of which are triphenylphosphonium (TPP)-based conjugates due to TPP’s strong mitochondrial targeting ability (Wang et al., 2017; Zielonka et al., 2017). In the last three decades, nanotechnology has captured great attention in medicine, and some nanoparticles have been investigated for mitochondrial targeting. The first nanomaterials applied for mitochondrial targeting were dequalinium (DQA) micelles (DQAsomes), which exhibit liposome-like self-assembly in aqueous solution (Weissig et al., 1998; Weissig and Torchilin, 2001; D’Souza et al., 2003). Then, other nanomaterials, including polymeric nanoparticles, metallic nanoparticles, and liposomes, were prepared as mitochondrial drug delivery carriers (Zhang and Zhang, 2016a; Wang et al., 2017).

Direct drug-targeting ligand conjugation is simple and easy to control, and the drugs can readily reach the mitochondria; however, the conjugation procedure can diminish the therapeutic effects and solubility of the drugs. In the case of the nanocarrier system, there is no concern regarding a loss of therapeutic effect because the physical interaction and solubility issue would be solved, but optimization has remained a challenge due to the use of many different compositions to prepare the nanocarrier, and mitochondrial delivery may also be delayed due to the nanocarrier features.

## Mitochondrial Targeting Ligand-Anticancer Drug Conjugates

Direct conjugation of therapeutic agents to molecules to enhance their physicochemical and biopharmaceutical efficiency results in a prodrug. In most cases, the prodrug is inactive until it is metabolized. The most successful examples are antibody-drug conjugates that can be activated by enzymatic cleavage after reaching the target site (Lambert and Morris, 2017). To produce a successful prodrug, selection of the linkage plays an important role. Generally, the linkages (bonds) are stimulus-responsive, for example, responsive to an enzyme, pH, temperature, or irradiation. Common bonds for prodrug formation are the amide bond, which is main bond in protein and peptide formation in living system and is cleavable by the protease enzyme; the hydrazone bond, which is degradable under acidic conditions; the ester bond, which is easily hydrolyzed in aqueous solution; the disulfide bond, which is cleavable by glutathione; and the ether bond, which is degraded under strong acidic or basic conditions.

In contrast, mitochondria-targeting ligand-anticancer drug conjugates can be active before and after cleavage. Therefore, the bond is not as important as in the prodrug, but it should be considered. A number of direct conjugates have been reported for mitochondrial delivery of anticancer drugs using various targeting moieties, including peptides [mitochondria-penetrating peptide (MPP), mitochondria-targeting sequence (MTS) peptide, and SS peptides] and lipophilic cations (TPP, rhodamine 123, and DQA) with various linkages. The most commonly used anticancer agents for mitochondrial delivery are doxorubicin (Dox), 5-fluorouracil (5-FU), paclitaxel, cisplatin, chlorambucil, porphyrin derivatives, and coumarin because those agents can be activated in mitochondria and exert therapeutic efficacy. In brief, Dox is an anti-cancer drug that intercalates into DNA and inhibits the progression of Topoisomerase II, leading to cell death. In addition, Dox derivatives have been known to interact with mitochondria by the disruption of major mitochondrial functions, damaging membrane-bound proteins and enzymes (Jung and Reszka, 2001). Cisplatin binds to nuclear DNA and induces DNA cross-linking and some studies confirmed that it can interact with mitochondrial DNA as well (Alexeyev et al., 2013). Paclitaxel derivatives are known to depolymerize microtubules and also some studies have shown that they can induce cytochrome c release by activating mitochondrial membrane proteins (D’Souza and Weissig, 2009). Chlorambucil is a DNA alkylating agent, which can alkylate both nuclear and mitochondrial DNA (Mourtada et al., 2013). Many porphyrin derivatives have been known to induce mitochondria functions in the presence of irradiation (Zielonka et al., 2017). Here, we compare various conjugates obtained from a variety of targeting ligands through different linkages, as well as the effect of the linker and targeting moiety for their activity. We summarize some specific properties of the mitochondria-targeted drug-conjugates in **Table 1**.

**Table 1:** Some specific characteristics of mitochondria-targeting ligands and anticancer drug conjugates.

Drug	Targeting ligand	Bond and spacer	Cell line and model	Exposure and IC_50_	Mitochondrial induction	Reference
Doxorubicin	TPP	Amide; propyl	MDA-MB-435/WT and MDA-MB-435/Dox	IC_50 for MDA-MB-435/WT and MDA-MB-435/Dox_: 0.31 and 21 μM	Caspase 3 activation and apoptosis	Han et al., 2014
Doxorubicin	MPP	Amide; succinate	HeLa cell, A2780, HL60	IC_50 for A2780 and A2780ADR_: 0.8 and 1 μM	ROS generation, TOPO II inhibition	Chamberlain et al., 2013a
F16	TPP	Butyl	U87MG, MDA-MB-231	IC_50 for U87MG_: 200 μM	Mitochondria uptake, ΔΨ decrease	Wu et al., 2014a
Chlorambucil	MPP	Amide	HeLa K562 xenograft mice	IC_50 for HeLa_: 3 μM MPP-Cbl dosage 15 mg/kg	Caspase 3, 7, 9 activated apoptosis; alkylate mtDNA, induce DNA lesions	Fonseca et al., 2011
Chlorambucil	TPP	Amide, propyl	MCF7 MIA PaCa-2 xenograft mice	IC_50 for MCF7_: 7 μM; dosage 10 mg/kg	Damaging the DNA, alkylates and cross-links DNA	Millard et al., 2013
Vitamin E	TPP	C-C; (-CH_2_-)_11_	HeLa and MCF7 cell; HCT116 tumor	IC_50 for HeLa and MCF7_: 0.44 and 1.9 μM	Bak protein mediated apoptosis	Dong et al., 2011
Porphyrin	TPP, TEA	Ether; butyl	MCF7	IC_50 for MCF7_ 1–3.2 μM	Photodynamic therapy; mitochondria destabilization	Lei et al., 2010
Porphyrin	Guanidine, Biguanidine, MLS peptide	Amide; peg for MLS	HepG2	IC_50 for HepG2_ of G-Por, BG-Por, MLS-Por: 4.8, 8.2, 9.8 μM	Photodynamic therapy	Sibrian-Vazquez et al., 2008
IR700DX-NHS	PS-6-TSPOmbb732	Urethane; valeric acid	TSPO-positive MDA-MB-231 and TSPO-negative MCF-7, MDA-MB-231 tumor-bearing mice	IC_50 for MDA-MB-231_: 5 μM at 60 min after laser	Apoptosis	Zhang et al., 2015a
BODIPY-phenylethynyl linker-F16	F16	C-C	SGC7901, A549, MCF7	IC_50 for SGC7901, A549, MCF7_: 2.49–6.20 μM	Apoptosis, decrease membrane potential, increase ROS	He et al., 2015
Gamitrinib	Cyclic guanidinium	Amide	A431, MDA-MB-231, MCF7, U87MG, and H460 tumor bearing mice	IC_50 for A431, MDA-MB-231, MCF7, and U87MG_: 4, 17, 15.2, 12.4 μM	ROS scavenging, decrease the level of cytochrome c	Baracca et al., 2003
5-FU(5-Fluorouracil)	F16	Ester, amide, disulfide	GES-1, SGC-7901	IC_50 for GES-1 cell_ of F16-OOC-FU and 5-FU: 60 and 40 μM	Thymidylate, pyrimidine, DNA	Agemy et al., 2011
Coumarins	TPP	C-C	HeLa	IC_50 for HeLa_: 200 μM	ROS generation, MMP decrease, apoptosis	Wang and Xu, 2017
2,4-Dihydroxybenzaldehyde	TPP	Ether, hexyl	HeLa, MDA-MB-213	IC_50 for HeLa and MDA-MB-213_: 0.17–0.45 μM	Mitochondrial aggregation, MMP decrease, ROS generation	Hu et al., 2014
(KLAKLAK)_2_	TPP	Amide, butyl	KB, HeLa, and COS7	IC_50 for KB and HeLa cells_: 8.2 and 28.5 μM	Disrupt the mitochondrial membrane, activation of caspase 3, 9, cytochrome c release	Chen et al., 2013
Cisplatin	MPP	Amide	A2780WT and A2780 CP70	IC_50 for A2780WT and A2780 CP70_: 7.5 and 7.5 μM	Attachment of alkyl groups to DNA bases	Wisnovsky et al., 2013

### Peptide-Anticancer Drug Conjugates

Peptides are favorable molecules for medical application due to their biocompatibility in physiology. As targeting ligands, many peptides have been developed, and the most widely used ones are cell-penetrating peptides (5–30 amino acids) such as TAT (transactivator of transcription, GRKKRRQRRRPQ) (Vives et al., 2008). Most peptides have a positive charge, which promotes cell membrane penetration, and short sequences. For mitochondrial targeting, some peptides have been prepared and successfully applied based on the cell-penetrating peptide sequence. The mitochondria-targeting peptides (mitochondria-penetrating peptide, mitochondria-targeting sequence, SS peptide, and other peptides) were conjugated with various anticancer agents to improve their therapeutic efficacy.

#### Mitochondria-Penetrating Peptide (MPP)-Drug Conjugates

Kelley’s group developed a series of short peptides for mitochondrial targeting based on cell-penetrating peptides such as TAT (Horton et al., 2008; Yousif et al., 2009a; Jean et al., 2016). Her group introduced the hydrophobic cyclohexyl monomer between arginine molecules to improve its lipophilicity and further explored the mitochondrial targeting ability of sequences of short peptides (Horton et al., 2008; Yousif et al., 2009a). The peptides were attached to several anticancer drugs to assess whether the peptides could improve mitochondrial targeting and enhance the therapeutic effect. Chamberlain et al. (2013a) hypothesized that this approach could prevent drug resistance, which is the most crucial problem associated with current chemotherapy for many cancers.

**FIGURE 1:**
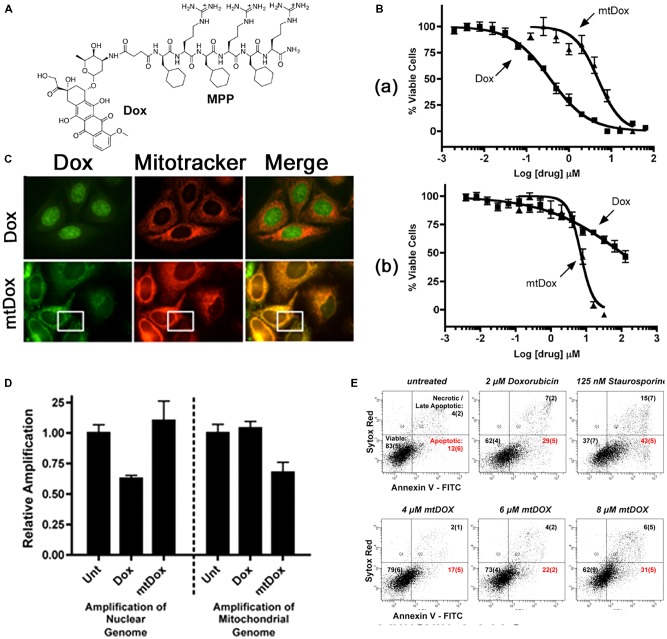
**(A)** Chemical structure of mtDox. **(B)** Cytotoxicity data. **(a)** mtDox and Dox are both toxic to sensitive A2780 cells. **(b)** The toxicity of Dox is highly attenuated in A2780ADR Pgp-expressing cells. **(C)** Intracellular localization of mtDox by confocal microscopy, Dox and mtDox (green channel) and Mitotracker 633 (red channel). **(D)** Assessment of DNA strand breaks in cells by measuring the relative amplification of nuclear and mitochondrial DNA segments. **(E)** Flow cytometry analysis of A2780 Annexin V-FITC (A-FITC)/SYTOX Red (SR) cell staining. Images were adapted with permission from reference (Chamberlain et al., 2013b).

Among several short peptides (L-cyclohexyl alanine-D-arginine)_3_ (F_x_,r)_3_ is known to hold promising mitochondria-targeting properties (Horton et al., 2008). Therefore, the peptide has been attached to Dox, cisplatin, and chlorambucil to explore its activity in drug-resistant cells (Fonseca et al., 2011; Chamberlain et al., 2013a; Mourtada et al., 2013; Wisnovsky et al., 2013). The peptide-Dox conjugate (mtDox) was prepared using an amide bond between the free amine group of Dox and carboxyl end of the short peptide (Chamberlain et al., 2013a). For Dox, it is well-known that Dox is pumped out by p-glycoprotein (Pgp) transporter in drug-resistant cells and also that Dox can be accumulated in lysosomes by lysosomal Pgp activity (Seebacher et al., 2016). Nevertheless, mtDox showed high accumulation in mitochondria compared with free Dox (**Figure 1**). Further studies have been carried out in drug-sensitive and resistant cells. Although mtDox has less cell killing effects than free Dox in drug-sensitive cells, it exhibited strong activity in drug-resistant cells (**Figure 1B**). The reason for this behavior is that mtDox (1) is not pumped out by p-glycoprotein (Pgp) transporter even as a substrate due to blockage of the free amine group of Dox, which is the reactive part required for Pgp-transporter binding, and (2) is selective for mitochondrial localization. These authors claimed that mtDox intercalated into mtDNA, and it is known that Dox functions by inhibiting DNA topoisomerase II protein and triggers apoptosis (Pommier et al., 2010).

In contrast, this peptide MPP-cisplatin conjugate (mtPt) was prepared to overcome cisplatin resistance in cancer cells because cisplatin, a widely used anticancer drug, is known to be resistant to various cancers (Wisnovsky et al., 2013). The activity of mtPt is quite similar in both sensitive and resistant cells [A2780 wild-type (WT) and cisplatin-resistant A2780 CP70 cell lines]. A subcellular uptake study showed that mtPt localized preferentially in mitochondria, and further study demonstrated that mtPt generated approximately two times higher levels of ROS than its free counterpart, damaging mtDNA and inducing apoptosis. Furthermore, through conjugation of the short peptide to chlorambucil to improve the therapeutic effect of chlorambucil, the MPP-chlorambucil (mtCbl) conjugate was also obtained and confirmed to localize in mitochondria due to the cyclohexylalanines and positive charge of the peptide sequence (Mourtada et al., 2013). Initial results showed that the conjugate was able to induce apoptosis, but a later study demonstrated that mtCbl led to necrotic cell death due to alkylation activity, which was confirmed by activation of caspase 3/7 and increases in protease activity (Mourtada et al., 2013). Among these conjugates, the most potent one is mtCbl, followed by mtDox and mtPt, compared to their counterparts.

#### Mitochondria-Targeting Sequence (MTS) Peptide-Drug Conjugates

Mitochondria-targeting sequence peptides consist of 20–40 amino acids that are recognized by receptors at the mitochondrial surface. Various translocases of the outer and inner membranes mediate the import and intramitochondrial sorting of MTS-containing peptides, which is driven by ATP or by the mitochondrial transmembrane potential (Yousif et al., 2009a). Multiple MTSs have been successfully applied for the mitochondrial delivery of chemically different cargos, including proteins, catalytically proficient enzymes and nucleic acids (Vestweber and Schatz, 1989; Srivastava and Moraes, 2001; Mukhopadhyay et al., 2005). The major drawbacks of this approach are associated with the large molecular size of MTSs and their solubility and intrinsically poor membrane permeability (Yousif et al., 2009a,b). Lin et al. (2015) conjugated MTS peptide (MLRAALSTARRGPRLSRLL), a well-known natural presequence from mitochondrial-oriented protein aldehyde dehydrogenase (ALDH), to a cell-penetrating peptide (R_8_) due to its cell internalization ability to enhance cell penetration. Three histidines were introduced into the conjugate to supply a buffering effect upon protonation of the imidazole ring inside endosomes or lysosomes, inducing rupture of endosomal/lysosomal membranes for effective release of the entrapped conjugate into the cytosol. The hydrophobic porphyrin molecule was also conjugated to the MLS peptide (MSVLTPLLLRGLTGSARRLPVPRAKIHSL, isolated from subunit VII of human cytochrome c oxidase), which is encoded in a commercially available expression vector (pShooter) that targets recombinant protein to the mitochondria of mammalian cells, in the presence of a short poly(ethylene glycol) (PEG) spacer (Sibrian-Vazquez et al., 2008). It showed high levels of cell uptake but low mitochondrial uptake. The high cell uptake might be related to the reduced aggregation tendency of the conjugate in aqueous media due to fewer intra- and intermolecular interactions. The conjugate showed less localization in mitochondria than in lysosomes because of the important roles of substituents of the porphyrin periphery in cellular uptake and subcellular distribution mechanisms (Horobin, 2002). In addition, since the conjugate localized extensively to lysosomes, it might exhibit poor endosome escape, resulting in low mitochondrial accumulation.

### Delocalized Lipophilic Cation (DLC)*-*Anticancer Drug Conjugates

Delocalized lipophilic cations are molecules that possess both lipophilicity and a positive charge. Therefore, they can easily pass through phospholipid membranes, plasma membranes, and mitochondrial outer membranes through diffusion. The delocalized positive charge allows the molecules to enter the matrix of mitochondria, which is highly negatively charged with a strong negative membrane potential (-180 mV), through the protein gradient process (Chen, 1988; Torchilin, 2006; Heller et al., 2012). Several DLCs are used as mitochondria-targeting ligands, such as TPP, rhodamine 123, DQA, guanidine, and F16 (Wang et al., 2010; Modica-Napolitano and Weissig, 2015; Zielonka et al., 2017). These mitochondria-targeting ligands can be linked to various anticancer agents through covalent bonds, such as amide, disulfide, ester, ether, and hydrazine bonds (**Table 1**). We discuss in more detail the conjugates and their specific results in the examples below.

#### Triphenyl Phosphonium (TPP)-Drug Conjugates

**FIGURE 2:**
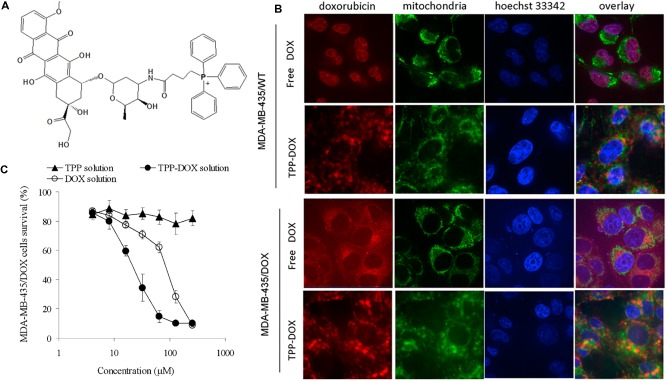
**(A)** Chemical structure of TPP-DOX. **(B)** Subcellular localization and **(C)** cytotoxicity of free DOX and TPP-DOX in drug-sensitive and resistant cancer cells; adapted with permission from reference (Han et al., 2014).

The most common example of a DLC is TPP, which is a positively charged phosphorus atom surrounded by three lipophilic phenyl groups that increase its combination. The TPP moiety has been employed as a probe to explore the mitochondrial membrane potential for more than 40 years (Lampidis et al., 1985). Consequently, its behavior and interaction with mitochondria are well-defined (Zhang and Zhang, 2016a). The relative TPP concentrations inside the negatively charged membrane compartments are increased by one order of magnitude for every 60 mV of negative membrane potential. Given the active transport of salt ions by membrane-bound pumps, the interior part of the plasma membrane is negatively charged compared with the exterior part. As a result, the plasma membrane potential generally ranges between -30 and -60 mV, which is sufficient to promote up to a 10-fold accumulation of TPP inside the cell. Many TPP-anticancer agent conjugates, including TPP-doxorubicin (Han et al., 2014), TPP-chlorambucil (Millard et al., 2013), TPP-F16 (Wu et al., 2014a), TPP-vitamin E (Dong et al., 2011), TPP-therapeutic peptide (Chen et al., 2013), TPP-porphyrin photosensitizers (Lei et al., 2010; Hu et al., 2014; Zhang et al., 2015a), and TPP-coumarin (Wang and Xu, 2017), are known and were described in a recent review (Zielonka et al., 2017). Here, we briefly discuss and compare some specific examples. Han et al. (2014) synthesized TPP-doxorubicin (TPP-Dox) via the reaction between the carboxyl group of the TPP moiety and the amine group of Dox to deliver the drug into mitochondria with the aim of overcoming drug resistance (**Figure 2A**). A cell uptake study revealed that the TPP-Dox conjugate had lower uptake by MDA-MB-435/WT cells than free Dox, indicating that the TPP moiety affected cell membrane transport. In contrast, in MDA-MB-435/Dox cells, TPP-Dox showed much higher cell uptake than free Dox, supporting the possibility of TPP-Dox by-passage through P-glycoprotein and other pumps overexpressed in a resistant cell line. A subcellular localization study by confocal microscopy revealed that in drug sensitive cells, free Dox was mostly found in the nucleus (**Figure 2B**), while it was not found in the nucleus in drug-resistant cells. In contrast, in both cell lines, the TPP-Dox conjugate was not localized in the nucleus and accumulated extensively in mitochondria (**Figure 2B**). A further time-dependent cytotoxicity study showed that TPP-Dox had an approximately fourfold lower IC_50_ than free Dox in drug-resistant cells after 48 and 72 h of incubation (**Figure 2C**). These results implied that the TPP-Dox conjugate was able to overcome drug resistance, but it was difficult to apply it *in vivo* due to its low solubility. Similar studies have been reported using Dox-nitrooxy or 3-phenylsulfonylfuroxan conjugates in a human colon cancer cell line (Riganti et al., 2013).

Another example is provided by vitamin E (VE) analog conjugates with the TPP molecule (Dong et al., 2011). VE analogs are known to induce cell apoptosis by affecting mitochondrial complex II, where they affect ubiquinone (UbQ) as a natural electron acceptor via the succinate dehydrogenase activity of complex II during the conversion of succinate to fumarate (Neuzil et al., 2001a,b, 2007). As anticancer agents, VE analog α-tocopheryl succinate (α-TOS), vitamin E succinate (VES), and VE were conjugated to the TPP moiety. Among the conjugates, MitoVES, which possesses an 11-carbon linker between tocopheryl succinate and the TPP moiety, strongly induced apoptosis (more than 90%) in various cancer cell lines compared with the other conjugates and its counterpart. The reduced apoptotic activity of VES4TPP was due to blockade of the free carboxyl group, which is essential for the apoptogenic activity of the compound (Dong et al., 2011). In addition, MitoVES did not induce apoptosis in non-malignant cells. The IC_50_ values of MitoVES in various cancer cell lines were 10- to 30-fold lower than those of the non-targeted counterpart, whereas the IC_50_ of MitoVES was much greater in non-malignant cells than in malignant cells. Furthermore, flow cytometry analysis showed that MitoVES generated ROS. Greater mitochondrial localization of MitoVES was confirmed by confocal microscopy due to affinity of the positive TPP group to the high negative potential at the matrix face of the mitochondrial inner membrane (MIM). MitoVES is known to induce mitochondria-dependent apoptosis, which involves the transcriptional modulation of bcl-2 family proteins. Furthermore, an antitumor study revealed that MitoVES greatly suppressed tumor growth compared with α-TOS in tumor-bearing mice.

In addition, Millard et al. (2013) compared a TPP-chlorambucil conjugate with other similar cationic moiety-linked chlorambucil conjugates with regard to enhanced therapeutic activity. As mentioned previously, chlorambucil is an anticancer agent that damages DNA. The authors synthesized three different conjugates, TPP-, tributyl phosphonium (TBP)-, and tricyclohexylphosphonium (TCP)-chlorambucil, and one negative control, TPP-dihydroxychlorambucil, and screened the cell cytotoxicity in the MCF7 cancer cell line. The IC_50_ values of the conjugates were 100, 7, 35, 40, and 80 μM for free chlorambucil, TPP-chlorambucil, TBP-chlorambucil, TCP-chlorambucil, and the negative control, respectively. This result indicated that the presence of aryl rings resulted in a higher potency than the presence of saturated hydrocarbons. Moreover, a cytotoxicity study of free chlorambucil and TPP-chlorambucil was performed in 10 other cancer cell lines, and in all cases, the conjugate had a higher potency, with a sensitivity ratio ranging from 7.7 to 79.9. Next, they found that the conjugate accumulated extensively in mitochondria and caused mitochondrial DNA damage, leading to nitrogen mustard-induced cell death. An *in vivo* antitumor study revealed that the TPP-chlorambucil conjugate possessed greater tumor suppression efficiency compared with the free counterpart, but it could not completely suppress tumor growth.

**FIGURE 3:**
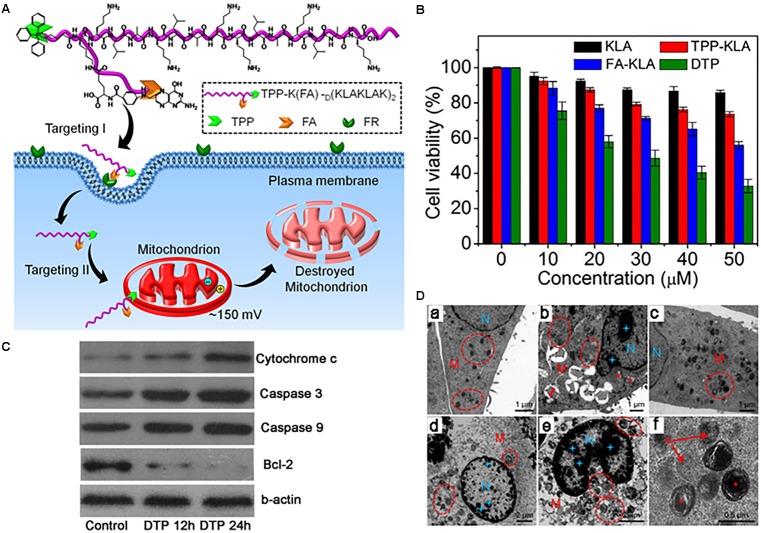
**(A)** Dual-targeting pro-apoptotic peptide (DTP) to selectively target cancer cells and specifically damage mitochondria to lead to programmed cell death. **(B)** Cell viability of folate receptor (FR) (+) HeLa cells incubated with KLA, TPP-KLA, FA-KLA, and DTP for 48 h. **(C)** Western blot analysis of apoptosis-related proteins in KB cells incubated with DTP for different times, using β-actin as the control. **(D)** TEM images of **(a)** KB cells incubated with no DTP, **(b,c)** KB cells incubated with DTP (20 mM) for 12 h, and **(d)** enlarged circled area in **(c)**. **(e,f)** KB cells incubated with DTP (20 mM) for 24 h. Mitochondria (M) are highlighted by red circles and red pentacles. Nucleus (N) is highlighted by blue doji stars. Images were adapted with permission from reference (Chen et al., 2013).

Chen et al. (2013) developed a dual-targeted pro-apoptotic peptide (KLAKLAK)_2_ for delivery into mitochondria and enhanced activity of the peptide. TPP was attached to the peptide via an amide bond, and folic acid was linked to the cell membrane-targeting ligand, enhancing cellular uptake of the peptide via receptor-mediated endocytosis (**Figure 3A**). The pro-apoptotic peptide is known to disrupt the mitochondrial membrane and induce mitochondria-dependent cell-free apoptosis without toxicity outside of cells (Ellerby et al., 1999; Agemy et al., 2011). To avoid degradation by proteases, D-amino acids were applied for the synthesis. The conjugate (IC_50_ 8.2 μM) showed much stronger cytotoxicity than folate-KLA peptide conjugate alone (IC_50_ 28.5 μM) in KB and HeLa cells (**Figure 3B**), indicating that TPP targeting assisted cytotoxicity. Further studies showed that the conjugate could activate cytochrome c and caspase 3/9 proteins in cancer cells, leading to apoptotic cell death (**Figure 3C**). In addition, TEM images of the cells confirmed that mitochondrial disruption occurred after the conjugate treatment (**Figure 3D**).

#### F16 ((E)-4-(1H-indol-3-ylvinyl)-*N*-methylpyridinium iodide)-Drug Conjugates

F16 is a delocalized cation and accumulates in the mitochondrial matrix. Initially, Xiang et al. (2013) prepared the F16-5-FU direct conjugate, but it reduced the efficacy of 5-FU. Therefore, they decided to use other linkers that are vulnerable bonds, including the ester bond, amide bond, and disulfide bond. Among the conjugates, F16-OOC-FU showed the strongest toxicity in the SGC-790 (human gastric carcinoma) cell line (Wang et al., 2016). They examined whether this toxicity was associated with cell uptake. The results illustrated that F16-OOC-FU and F16-SS-FU were internalized rapidly by the cells, whereas F16-5FU and F16-NHOC-FU were not internalized well, potentially due to lipophilicity. Enhanced cell uptake of F16-OOC-FU led to greater cytotoxicity, but F16-SS-FU did not affect cytotoxicity due to insufficient glutathione in the cytosol. In addition, the mitochondrial targeting moiety F16 was introduced to boron-dipyrromethene (BODIPY) via a phenylethynyl linker, and the resultant bifunctional mitochondria-targeting anticancer drug conjugate [F16-phenylethynyl-BODIPY conjugate (FPB)] with therapeutic and imaging properties were synthesized. When FPB was applied to cancer and normal cells, it was internalized to a greater extent by cancer cells (e.g., SGC-7901) than by normal cells (e.g., GES-1) (He et al., 2015).

#### Rhodamine-Drug Conjugate

Rhodamine derivatives are also mitochondria-targeting agents, given their binding affinity to mitochondrial membranes, which subsequently damages the electron transport chain (Baracca et al., 2003). Mechanistically, their mitochondrial accumulation is caused by their lipophilic and cationic properties, which aid in crossing the double mitochondrial membrane and remaining within the negatively charged mitochondrial matrix (Lampidis et al., 1985). Similar to rhodamine 123, rhodamine 19 is a potential mitochondria-targeting moiety (Antonenko et al., 2011). Using rhodamine, several reports have described antioxidant targeting to mitochondria, but very few have addressed anticancer agents. Rhodamine 123 itself has been studied for cancer treatment by conjugation or without conjugation to another molecule (Lampidis et al., 1985; Baracca et al., 2003; Antonenko et al., 2008, 2011). Despite testing in a clinical study for prostate cancer treatment, the dosage (92 mg/m^2^) was not tolerable, and thus, the efficiency was insufficient.

#### Guanidine-Drug Conjugates

**FIGURE 4:**
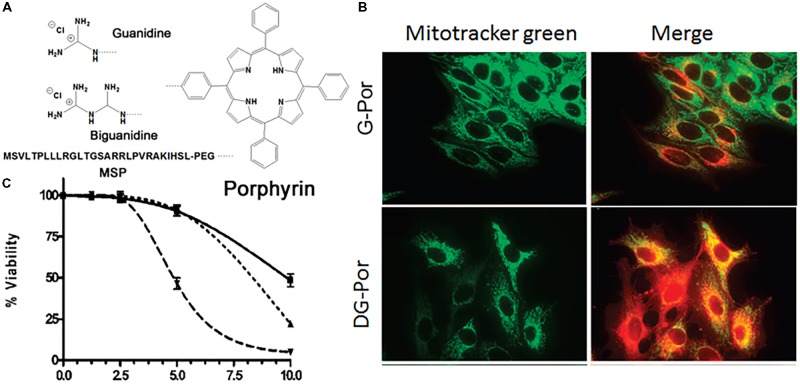
**(A)** Chemical structures of guanidine-porphyrin (G-Por), diguanidine-porphyrin (DG-Por), and peptide-porphyrin conjugates; **(B)** mitochondrial localization of G-Por and DG-Por in HepG2 cell at 24 h (green for MitoTracker^®^ green and red for porphyrin); **(C)** phototoxicity of G-Por (dashed line), DG-Por (dotted line), and peptide-Por (continuous line) toward HepG2 cells using a 1 J/cm^2^ dose of light. Images **(B,C)** were adapted with permission from reference (Sibrian-Vazquez et al., 2008).

Guanidinium and biguanidinium, which hold delocalized positive charges, were conjugated to a hydrophobic porphyrin molecule, which is a photosensitizer (PS) and phototoxic agent, to improve mitochondrial accumulation through the high membrane potential across the inner mitochondrial membrane for the treatment of cancer (**Figure 4A**) (Sibrian-Vazquez et al., 2008). A cellular uptake and subcellular localization study revealed that guanidine-porphyrin (G-Por) as readily taken up by cells and accumulated in mitochondria and the ER compared with biguanidine-porphyrin (BG-Por), which was mainly localized in cell membranes, lysosomes, and mitochondria, associating with their structure and their ability to interact with negatively charged groups in the cell membrane (**Figure 4B**). The G-Por conjugate showed 1.8-fold higher phototoxicity than the BG-Por conjugate, which may be related to the difference in cellular uptake and localization (**Figure 4C**). In addition, the guanidine group is a planar structure that acts as a bidentate ligand, but the biguanidine molecule is non-planar due to the requirement to reduce the allylic strain. Partial mitochondrial localization of G-Por and BG-Por could allow the association of guanidine and biguanidine groups, which play important roles in the lysosome/endosome escape of conjugates through membrane rupture, probably by the proton sponge effect, allowing the PS to be distributed to other organelles. Kang et al. (2009) conjugated Gamitrinib, an Hsp90ATPase inhibitor, in human mitochondria to four kinds of cyclic guanidinium. Monomers, dimers, trimers and tetramers of cyclic guanidinium were introduced to Gamitrinib. Gamitrinib-G1, Gamitrinib-G2, Gamitrinib-G3, and Gamitrinib-G4 were synthesized. Among them, Gamitrinib-G4 and Gamitrinib-G3 exhibited increased mitochondrial accumulation and 6- to 10-fold increased anticancer efficacy in cancer cells compared with Gamitrinib-G1 and Gamitrinib-G2 due to different membrane depolarization activities.

## Mitochondria-Targeted Nanocarriers

Although direct conjugation showed promising results for mitochondrial targeting, some challenges persisted in terms of the solubility and clinical application of the therapeutic agents due to poor biocompatibility. Therefore, the use of nanocarriers can enhance the potential of therapeutic agents. Nanotechnology in medicine has captured broad attention over the last three decades due to the large specific surface area, easy functionality, drug-loading capacity, and biocompatibility (Bogart et al., 2014; Wicki et al., 2015). In contrast, disorders such as cancer allow the selective accumulation of nanoparticles in the target area. To date, several kinds of nanocarrier systems, including polymeric nanoparticles, liposomes, micelles, metallic nanoparticles, carbon nanoparticles, and dendrimers, have been developed in medicine, particularly in the drug delivery field. Nanocarrier systems must have some optimal features, such as an average size of 10–200 nm that allows the EPR effect, a neutral surface charge, high biocompatibility, and reduced toxicity. In the case of mitochondrial targeting, the size of nanocarriers should be small as possible. In most cases, a nanoparticle is attached to a targeting ligand to deliver it to mitochondria, but in a few cases, the nanoparticle itself can be the targeting agent to the mitochondria due to its properties. Some physical and biological properties of drug-loaded nanoparticles are summarized in **Table 2**.

**Table 2:** Some physical and bioactivity characteristics of mitochondria-targeting drug-loaded nanoparticles.

Nanocarrier	Drug	Targeting moiety	Size and zeta potential	Models	Exposure and IC_50_	Mitochondrial induction	Reference
Poly(𝜀-caprolactone)	Dox and Dox-HCl	TPP	50 nm; 40 mV	HeLa and HepG2	IC_50-HeLa and HepG2_: 0.007–3.2 and 0.009–3.2 μg/mL	Apoptosis	Cho et al., 2015
Chitosan-stearic acid micelle	Celastrol	TPP-PEG	63.5 nm; 22.1 mV	MCF7 and A549; MCF7-tumor bearing nude mice	IC_50-MCF7 and A549_: 1.81 and 0.76 μg/mL	ROS generation, cytochrome c release induced apoptosis	Tan et al., 2018
Liposome (TPGS)	Topotecan	DQA	64 nm; -0.54 mV	MCF7 and MCF7/ADR; MCF7/ADR bearing nude mice	IC_50 for MCF7 and MCF7/ADR_: 2.04 and 1.13 μM; Topotecan dosage 5 mg/kg	ΔΨ decrease; cytochrome c release- induced apoptosis	Yu et al., 2012
Liposome (TPGS)	PTX	TPP	84 nm; 1.93 mV	A549 and A549/cDDP	IC_50 for A549 and A549/cDDP_: 0.05 and 0.7 μM	Cytochrome c release mediated apoptosis	Zhou et al., 2013
DQAsomes and DQA80s	DQAsome	DQA	208 nm; 56.3 mV and 203 nm; 60.2 mV	U373-MG; HeLa	IC_50 for U373-MG and HeLa_: 35 and 55 μg/mL	ROS generation, membrane destabilization, MAPK signal activation apoptosis	Bae et al., 2018
DQA80plexes	pDNA	DQA	444 nm; 17.1 mV	HeLa	–	ΔΨ decrease due to DQAsome	Bae et al., 2017
TPP-Lonidamine PEG micelle	Dox	TPP	110 nm; 0.7 mV	MCF7 and MCF7/ADR; MCF7 bearing nude mice	IC_50 for MCF7 and MCF7/ADR_: 2 and 4 μg/mL; Dox dosage 10 mg/kg	ROS generation, membrane potential decrease, caspase 3, 9-activated apoptosis	Liu et al., 2017
PLGA-*b*-PEG NPs	ZnPC	TPP	65–75 nm; 24–34 mV	MCF7 and HeLa	IC_50 for HeLa and MCF7_: 96 and 49 nM	Early stage apoptosis	Marrache et al., 2013
DSPE-PEG micelle	α-TOS-Dox, α-TOS-cisPt, α-TOS-PTX	α-TOS	163, 186.3, 167.9 nm	HeLa	IC_50 for HeLa_: 6.8, 7.5, 28.1 μM	Cytochrome c release-mediated apoptosis, DNA and tubulin damage	Mallick et al., 2015
Peptide nanofiber	Dox	DDDK peptide	46 nm; negative	HeLa and U87MG	IC_50 for HeLa_: 400 nM	ENTK enzyme targeted cell death	He et al., 2018
Peptide polyoxometalates NPs	-	Dmt-D-Arg-Phe-Lys-NH2	60 nm; -13.2 mV	MCF 7	-	Mitophagy-induced cell death	Zhang et al., 2015b
D-Alpha-Tocopheryl PEG succinate-CQDs	Dox	TPP	101.4 nm; 21.04 mV	MCF7 and MCF7/ADR	IC_50 for MCF7/ADR_: 8.45 μg/mL	ΔΨ decrease; apoptosis	Zhang et al., 2017
TPP-Coumarin NPs	Dox	TPP	20 nm; -17.5 mV	HeLa, HCT116, A549, COV 434	-	Mitochondrial dysfunction	Lee et al., 2018
Graphene oxide	Dox	Glycyrrhetinic acid	200 nm; -37.6 mV	HepG cell and HepG-bearing nude mice	IC_50 for HepG_: 0.27 μM; Dox dosage 6 mg/kg x	Caspase 3,7,9-induced apoptosis	Zhang et al., 2018
Graphene oxide	PheoA	Positive charged Nps itself	100–400 nm; positive charge	U87MG and MCF7	IC_50 for U87MG_: 1 μg/mL	ΔΨ decrease; apoptosis	Wei et al., 2016
Gold NPs	SMI#9	SMI#9	40 nm; -16.2 mV	MDA-MB-468, HCC1937 TNBC	IC_50 for MDA-MB-468 and HCC1937_: 10 μM	Rad6-inhibition induced apoptosis	Haynes et al., 2016
TPP-Dox-Hyaluronic acid NPs	Dox	TPP	192 nm; -28.8 mV	MCF7/ADR cell and MCF7/ADR-tumor bearing mice	IC_50 for MCF7/ADR_: 15 μg/mL; Dox dosage 4 mg/kg x 9	Apoptosis	Liu et al., 2018
PAMAM dendrimer	TPP	TTP-PEG	6–12 nm; 14–53 mV	A5494	IC_50 for A549_ of G4NH_2_-10TPP and G4NH_2_-0TPP: 2.95 μM and over 20 μM	–	Young et al., 2018

### Polymeric Nanoparticles and Micelles

Polymeric nanoparticles are frequently prepared from biodegradable amphiphilic polymers. The advantages of polymeric nanoparticles can be biocompatibility, a small size, high drug loading capacity, good water solubility, low toxicity, and easy modification (Kedar et al., 2010; Oerlemans et al., 2010; Yokoyama, 2010; El-Say and El-Sawy, 2017). Some examples of polymeric nanoparticles for mitochondria-targeted drug delivery are poly(ethylene glycol) (Khatun et al., 2017), poly(𝜀-caprolactone) (PCL) (Cho et al., 2015; Choi et al., 2017), polysaccharides (e.g., chitosan, hyaluronic acid, and dextran) (Kim et al., 2016; Hou et al., 2017; Liu et al., 2018; Tan et al., 2018), poly[(2-hydroxypropyl)-methacrylic acid] (pHPMA) (Sun et al., 2017), poly(lactic-co-glycolic acid) (Marrache and Dhar, 2012; Marrache et al., 2013), and micelles formed from small amphiphilic molecules (He et al., 2018; Lee et al., 2018; Young et al., 2018).

**FIGURE 5:**
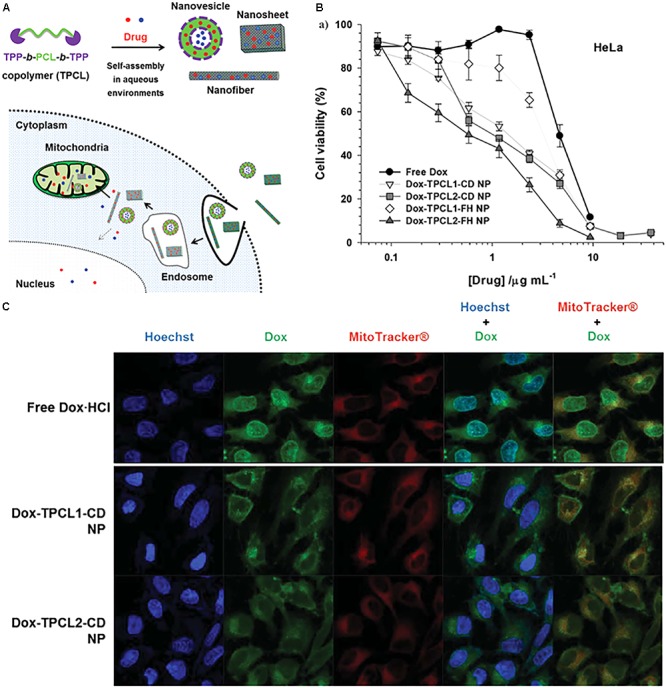
**(A)** Illustrated design concept of drug-loaded TPCL nanoparticles (NPs) in cells. The red solid circle (∙) and blue solid circle (∙) indicate hydrophobic and hydrophilic chemical drugs, respectively. **(B)** *In vitro* antitumor effects of Dox-loaded TPCL NPs in HeLa cells. **(C)** Intracellular distribution of free drugs and drug-loaded TPCL NPs and Dox-TPCL-CD NPs with [Dox] = 1 μg mL^-1^ in HeLa cells at 4 h post-treatment. Images were adapted with permission from reference (Cho et al., 2015).

The attachment of mitochondrial targeting ligands to polymeric nanoparticles has been widely explored for the delivery of therapeutic molecules to their active site. The concept of using polymeric nanoparticles for mitochondrial targeting is to deliver therapeutic molecules with reduced toxicity and to improve the biocompatibility of drugs. Polymeric nanoparticles are biocompatible and can carry a large number of cargo materials simultaneously to the target site due to their high loading capacity. Several kinds of polymeric nanoparticles have been applied to mitochondrial targeting, such as PCL with a low molecular weight, chitosan, dendrimers, liposomes, gold nanoparticles, and graphene oxide (Yu et al., 2012; Bielski et al., 2015; Cho et al., 2015; Haynes et al., 2016; Wei et al., 2016; Choi et al., 2017; Tan et al., 2018). Our group developed various TPP-conjugated PCL NPs and demonstrated their mitochondrial targeting ability in cancer cell lines (Cho et al., 2015; Choi et al., 2017; Khatun et al., 2017). We attached the TPP moiety at both ends of low-molecular-weight PCL polymers (0.5, 1.25, and 2 kDa). These polymers self-assemble in an aqueous environment with or without a hydrophobic anticancer drug (**Figure 5A**). The mean diameters of these NPs are approximately 100 nm, which is an optimal size for a drug carrier. As shown in **Figures 5B,C**, Dox-loaded PCL NPs preferentially accumulated in the mitochondria of HeLa cells compared to free Dox, resulting in a 3- to 5-fold higher cytotoxicity compared with free Dox. Additionally, the PCL polymeric nanoparticles were able to deliver both hydrophobic and hydrophilic Dox (Dox-HCl) into mitochondria. TPP-related cytotoxicity was observed due to its positive charge. These results support the potential of the polymeric carrier system in mitochondria-targeting drug delivery approaches.

Liu et al. (2017) described Dox-loaded TPP-lonidamine self-assembled nanoparticles for mitochondria-targeted cancer treatment). They also attached PEG to the NPs to enhance stability in the blood. The NPs have a size of approximately 110 nm and a neutral surface charge. As claimed by the authors, the NPs showed slow cellular uptake compared with Dox, but they were preferentially localized in mitochondria. Furthermore, the NPs showed greater cytotoxicity in both drug-sensitive and drug-resistant cancer cells compared with the other controls including free Dox. The results confirmed that the NPs generated high ROS levels, decreased the mitochondria membrane potential, and activated caspase 9 and caspase 3 proteins, which induce apoptosis. In addition, the NPs had a 12-fold higher half-life than free Dox in blood and suppressed tumor growth by 2- to 4.5-fold more than in the other groups. Interestingly, the authors claimed that the introduction of PEG to the NPs did not affect their cellular uptake and anticancer efficacy.

Tan et al. (2018) prepared a TPP-attached chitosan-stearic acid conjugate (TPP-CSOSA) loaded with celastrol (Cela), which is a weakly acidic anticancer drug, for drug delivery to mitochondria in a pH-dependent manner. The TPP-CSOSA micelles had a size of 86 nm, and Cela-loading reduced the size to 63 nm. They used a weakly acidic drug, which could easily dissociate in an alkaline pH environment such as the mitochondrial matrix (pH 8). The confocal data showed that FITC-labeled TPP-CSOSA localized in mitochondria in a time-dependent manner. The intracellular localization of NPs was analyzed by TEM imaging and validated their presence in mitochondria. Further studies have confirmed that NPs have greater cytotoxicity in cancer cells than the control groups and generate 3.2-fold higher ROS, which leads to a decrease in membrane potential and depolarization. *In vivo* studies have revealed that TPP-CSOSA/Cela NPs accumulate to a great extent in tumor tissues and have tumor suppression efficacy. The attachment of TPP to NPs enhanced cell uptake and tumor accumulation, and the use of the weakly acidic drug Cela led to rapid release at a higher pH, resulting in improved pharmacological action. Liu et al. (2018) prepared a hyaluronic acid-Dox-TPP conjugate using a hydrazone linkage to specifically deliver TPP-Dox to mitochondria. The conjugate formed nanoparticles with a size of 192 nm and a negative surface charge. A cell uptake study showed greater mitochondrial accumulation of the NPs in MCF/ADR cells, and further cytotoxicity and antitumor studies confirmed the enhanced efficacy compared with free Dox and TPP-Dox conjugates. Bielski et al. (2015) described a TPP-poly(amidoamine) dendrimer with different numbers of TPP showing different mitochondria-targeting behaviors. The TPP moiety was attached to dendrimers as a direct amide linkage or using the PEG spacer. Both direct conjugation and conjugation with the PEG spacer resulted in enhanced mitochondria-targeting behavior compared with the non-targeted dendrimers. In the case of direct conjugation, there was a direct correlation between mitochondrial targeting and the TPP density in dendrimers, while in the TPP-PEG dendrimer, mitochondrial targeting was not affected. Another example of the use of PEG for mitochondria-targeting NPs was reported to deliver pheophorbide a (PheoA) to mitochondria in the presence of TPCL (Choi et al., 2017). The data showed that the nanoparticle delivered PheoA to mitochondria, but the cell killing effect of free PheoA was not achieved.

**FIGURE 6:**
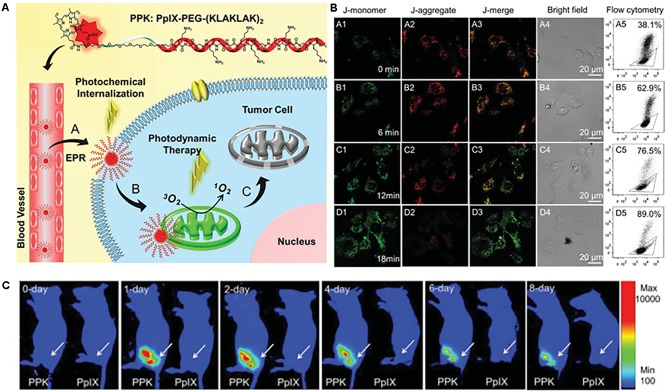
**(A)** Structure of PPK and schematic diagram of the mitochondria-targeted self-delivery process. Passive targeting to tumor cells and the photochemical internalization (PCI) effect enhanced cellular internalization of PPK under short-duration light irradiation. **(B)** Mitochondria-targeting mediated by (KLAKLAK)_2_. **(C)** ROS formation, mitochondrial destruction, and cell death mediated by the synergistic effect between (KLAKLAK)_2_ and PpIX under long-duration light irradiation. **(B)** CLSM images and the corresponding flow cytometric analysis of the mitochondrial membrane potential for HeLa cells when the cells were incubated with 40 μg mL^-1^ of PPK and then exposed to different irradiation durations: **(A1–A5)** without light irradiation (control); **(B1–B5)** with 6-min light irradiation; **(C1–C5)** with 12-min light irradiation; **(D1–D5)** with 18-min light irradiation. For **(A5–D5)**, the triangle separates populations with high (inside the triangle) and low (outside the triangle) membrane potential. **(C)** Intratumoral fluorescence distributions of PpIX and PPK on the 10th day after subcutaneous injection. Images were adapted with permission from reference (Han et al., 2015).

Han et al. (2015) linked porphyrin (PpIX), a clinically available photosensitizer, to PEG-proapoptotic peptide (KLAKLAK)_2_ to obtain a mitochondria-targeted photodynamic agent (**Figure 6A**). The conjugate was self-assembled and formed nanomicelles with a size of 91 nm. The micelle was found to decrease the mitochondrial membrane potential (**Figure 6B**), leading to cytochrome c release and apoptotic cell death. Animal studies showed that the micelles accumulated in the tumor area for 8 days (**Figure 6C**) and strongly suppressed tumor growth.

### DQAsome and Liposomes

DQAsomes were early nanocarriers for mitochondria targeting consisting of a bolaform self-assembly of DQA in aqueous solution due to the presence of a positively charged head group and alkyl chain (Weissig et al., 1998; Weissig and Torchilin, 2001; Weissig, 2015). The DQAsomes were incorporated with pDNA, antioxidant agents, and anticancer agents in the early 2000 (Weissig et al., 1998; Weissig and Torchilin, 2001; D’Souza et al., 2003), but they exhibited a low transfection efficiency and reduced ability to escape from endosomes, raising difficulties in the delivery their cargos to target site (D’Souza et al., 2003). In addition, they showed high cell killing activity in cancer cells compared with normal fibroblasts due to the disruption of mitochondrial transmembrane potential, blockade of ATP synthesis, and ROS generation (Sancho et al., 2007; Pajuelo et al., 2011). Therefore, many studies have focused on DQA-liposomes as a carrier (Yu et al., 2012; Zhou et al., 2013; Bae et al., 2017, 2018). Bae et al. (2017, 2018) developed DQA-liposomes consisting of DQA/DOTAP/DOPE or DQA80s (80% DQA) that can deliver the therapeutic agent to mitochondria and show anticancer effects. The liposomes can escape from lysosomes due to the usage of DOPE, which is a neutral lipid membrane disruption lipid. A cytotoxicity study revealed that DQA80s had higher activity in cancer cells than DQAsomes. A subcellular study showed that DQA80s accumulated to a high extent in mitochondria than simple DQAsomes. The TEM study then confirmed the intracellular localization of DQA80s. Furthermore, DQA80s were incorporated with pDNA and prepared DQAplexes (Bae et al., 2017). The liposome had a size of approximately 400 nm and a positive surface charge.

Drug-encapsulated DQA-based liposomes have been reported to specifically deliver drug to mitochondria (Wang et al., 2011; Yu et al., 2012; Zupancic et al., 2014). Topotecan hydrochloride, a derivative of camptothecin and a topoisomerase I inhibitor, was loaded into DQA and TPGS liposomes (Yu et al., 2012). Mitochondria-targeting topotecan-loaded DQA liposomes showed 2- and 4-fold higher cytotoxicity in MCF7 and MCF7/ADR cells, respectively, than free topotecan. The colocalization study revealed that the liposomes delivered the cargo to mitochondria at levels 21.2-fold higher than the free form and 12.9-fold higher than non-targeted liposomes by flow cytometry. A further study confirmed that liposomes could release cytochrome c following apoptotic cell death. An *in vivo* biodistribution study showed that Cy7-labeled DQA-liposomes accumulated in tumors, and a further antitumor study in MCF7/ADR tumor-bearing mice demonstrated that the DQA-liposomes strongly suppressed tumor growth compared with the other groups.

### Inorganic Nanoparticle Carriers

A number of nanoparticles have been studied for drug delivery, imaging, and therapeutic purposes, such as graphene oxide (GO), gold nanoparticles, silica nanoparticles, quantum dots, and carbon nanospheres, because of their biocompatibility, optimal size, large surface area, easy functionality, and specific physical properties (Wilczewska et al., 2012; Mattoussi and Rotello, 2013). Some mitochondria-targeting nanoparticles that were recently developed include GO (Wei et al., 2016; Zhang et al., 2018), lipid-coated carbon-quantum dots (Zhang et al., 2017), carbon-silica nanoparticles (Wang et al., 2018), polyoxometalates (Zhang et al., 2015b), and gold nanoparticles (Haynes et al., 2016) to deliver the therapeutic agent to mitochondria and overcome drug resistance. NPs have been known to successfully deliver anticancer agents to mitochondria with or without a targeting ligand. Some specific characteristics of nanoparticles are included in **Table 2**.

**FIGURE 7:**
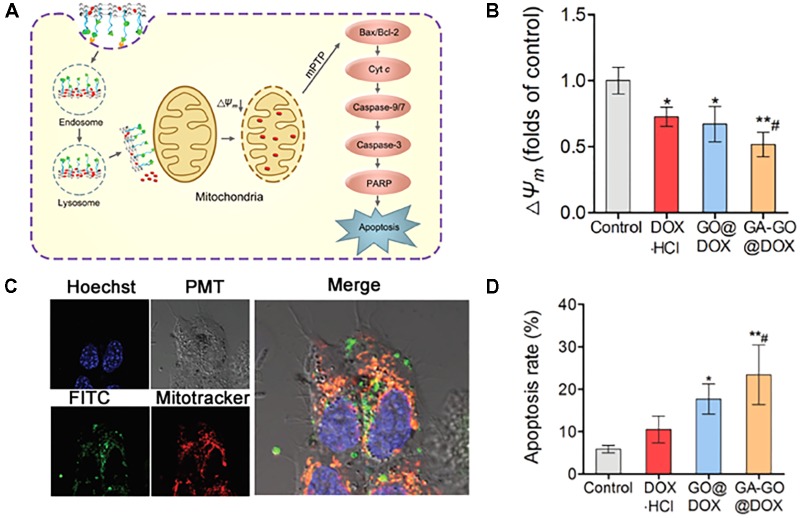
**(A)** Schematic illustration of the proposed mechanism of the MMA pathway of GA-GO@DOX. **(B)** ΔΨm of HepG2 cells after incubation with DOX⋅HCl, GO@DOX, GA-GO@DOX, and the control at an equivalent DOX dose of 0.5 μg mL^-1^ for 24 h. **(C)** CLSM images of the intracellular localization of GA-GO-FITC in HepG2 cells after treatment with GA-GO-FITC (800 μg mL^-1^) for 6 h. The cells were stained with organelle trackers for 45 min. **(D)** The apoptosis rate of HepG2 cells after treatment with DOX⋅HCl, GO@DOX, GA-GO@DOX, and the control. Images were adapted with permission from reference (Zhang et al., 2018). Bars represent means ± SD (*n* = 3); ^∗^*p* < 0.05 and ^∗∗^*p* < 0.01, compared to control; ^#^*p* < 0.05, compared to DOX⋅HCl.

Zhang et al. (2018) described glycyrrhetinic acid (GA)-attached GO with Dox as a model drug for mitochondria-targeted cancer treatment (**Figure 7A**). The authors chose GA for dual targeting to mitochondria and the cell membrane due to its ability to interact with the mitochondrial respiratory chain and binding affinity to protein kinase C (PKC) α, which is overexpressed on some cancer cells (Salvi et al., 2003; Fiore et al., 2004; Stankovich et al., 2006; Wu et al., 2008). In addition, GO is known to have a large specific surface area, high stability, functionality, and an optimal size for drug delivery (Yang et al., 2013; Battogtokh and Ko, 2016). Cell uptake was found to be PKC-dependent via the blockade of GA. Moreover, the NPs were confirmed to be able to reduce the membrane potential and activate caspases 3 and 9, resulting in apoptotic cell death (**Figure 7B**). The results showed that Dox-loaded GA-GO had twofold high cytotoxicity than free Dox in HepG2 cell, which was confirmed by robust uptake by cells and mitochondria (**Figure 7C**). The authors found that the NPs accumulated to a high extent in tumor tissue and liver due to the GA-targeting affinity, leading to apoptosis-mediated death (**Figure 7D**). Another example is the GO-photosensitizer complex, which has been used for mitochondria-targeted cancer therapy (Wei et al., 2016). The authors claimed that the nanoparticle preferentially accumulated in mitochondria compared with free PS due to the strong electronic interactions of the polarized GO with the mitochondria membrane and endosome-escape properties via simple traversal of the lipid membrane (Zhou et al., 2010; Wu et al., 2014b). Further studies have revealed that GO-PS has greater phototoxicity and induces apoptotic cell death.

Zhang et al. (2017) prepared mitochondria-targeting Dox-loaded carbon quantum dot (CQD) nanoparticles to overcome drug resistance. D-α-Tocopherol polyethylene glycol succinate (TPGS), an inhibitor of Pgp, was included in the nanoparticle to inhibit Pgp expression in drug-resistant cancer cells. CQDs are used as fluorescent probes for bioimaging, biolabeling, and biosensing due to their strong and stable fluorescence and low toxicity (Lim et al., 2015). TPP was conjugated to TPGS and covered the CQDs. The cytotoxicity results revealed that the Dox-loaded CQD nanoparticles had a fivefold lower IC_50_ value in drug-resistant MCF7 cells compared with free Dox. A further intracellular study revealed preferential mitochondrial localization of the NPs in drug-resistant cells. Mechanistic studies are currently outstanding.

Additionally, Zhang et al. (2015b) developed peptide-polyoxometalate (POM) nanoparticles as mitochondria-targeted anticancer agents. POM nanoparticles have photoluminescence properties, but their rapid quenching effect does not allow cell imaging. As a mitochondria-targeting agent, the positively charged short peptide Dmt-D-Arg-Phe-Lys-NH_2_ was chosen due to its selective targeting to mitochondria (Zhao et al., 2004). Preferential mitochondrial localization of the nanoparticle was demonstrated by confocal and TEM imaging. The authors claimed that the NPs could induce mitophagy through a Parkin (Parkinson juvenile disease protein 2)-mediated process.

Wang et al. (2018) described pyruvate-based mitochondria-targeting lipid-membrane coated silica-carbon (LSC) hybrid nanoparticles to solve the problem of drug resistance. These nanoparticles could produce ROS through near-infrared (NIR) laser irradiation. During preparation of the nanoparticles, pyruvaldehyde groups were formed with a structure very similar to pyruvate, which can specifically bind to the monocarboxylate transporters on the outer mitochondrial membrane (Pinheiro et al., 2012; Wen et al., 2013). Therefore, pyruvaldehyde could be a mitochondrial targeting moiety for this nanoparticle. Dox was encapsulated in this nanoparticle, and its effects on drug-sensitive and drug-resistant cancer cells were evaluated. Mitochondrial accumulation of the Dox-loaded NPs was demonstrated by confocal microscopy and TEM imaging. Pyruvate-mediated mitochondrial uptake was tested by pre-treatment with pyruvic acid, followed by a 40 to 3% decrease in mitochondrial uptake. A further cytotoxicity study showed that Dox-LSC nanoparticles with laser irradiation had a great killing effect in drug-resistant cancer cells compared with free Dox and LSC NPs. *In vivo* biodistribution and antitumor studies revealed that LSC NPs preferentially accumulated in tumor tissue and completely suppressed tumor growth. These results were due to the ability of the LSC NPs to target mitochondria and produce ROS under NIR irradiation to oxidize NADH to inhibit the production of ATP, thus reducing the function of efflux pumps and overcoming the drug resistance.

### Mitochondria-Targeted Drug Conjugate-Loaded Nanoparticles

In most cases, mitochondria-targeting ligand-drug conjugates are poorly water soluble, and thus they are difficult to apply in preclinical and clinical studies. One solution is to use a carrier system and deliver them to a target site such as a tumor. The conjugates could subsequently be released from the nanocarrier and reach the mitochondria. Several studies, including studies of TPP-docetaxel conjugates and TPP-photosensitizer-loaded albumin nanoparticles (Battogtokh and Ko, 2017; Battogtokh et al., 2018), paclitaxel, cisplatin or Dox-α-TOS conjugate-loaded lipid nanoparticles (Mallick et al., 2015), and DQA-Dox-loaded pH-responsive micelles (Song et al., 2015), have revealed dual-targeting to the tumor area and mitochondria. The directed targeting of nanosized materials could show delayed entry into mitochondria due to their size, but small mitochondria-targeting molecules can easily enter mitochondria. Therefore, the role of nanocarriers could be to deliver small molecules to the tumor area and cellular cytosol.

Butyl carboxyl TPP was conjugated to docetaxel (DTX) via an ester bond and loaded into folate-targeted albumin nanoparticles to enhance the therapeutic activity of DTX (Battogtokh et al., 2018). Albumin was coupled to the cholesteryl anchor to form self-assembled nanoparticles, which is known to have a high drug loading capacity and a size of approximately 120 nm. Thus, the TPP-DTX conjugates were loaded with cholesteryl-albumin and evaluated *in vitro* and *in vivo*. The results revealed that the conjugate accumulated in mitochondria and induced apoptosis-mediated cell death. In contrast, TPP-photosensitizer conjugate-loaded albumin nanoparticles were prepared to localize the photosensitizer in mitochondria because they must be localized at the specific target site due to the short half-life of ROS generated from PS through laser irradiation (Battogtokh and Ko, 2017). If the ROS are generated at a site that is distant from the target site, the therapy will fail due to insufficient damage. Some PS are known to accumulate in mitochondria, but they are also localized in other organelles. Therefore, there is a need to selectively deliver PS to mitochondria. TPP-PS conjugate-albumin NPs showed preferential mitochondrial accumulation, mitochondrial membrane destabilization, apoptotic cell death, and enhanced antitumor efficacy in tumor-bearing nude mice.

**FIGURE 8:**
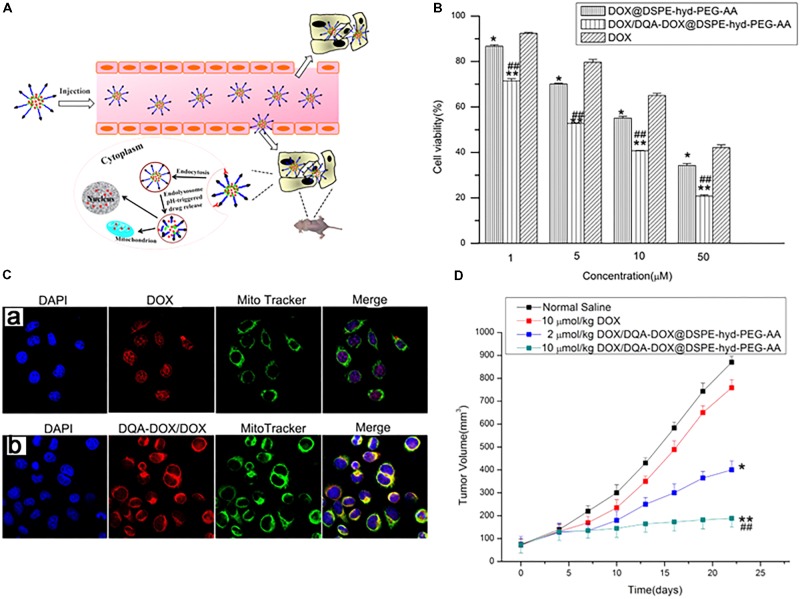
**(A)** Schematic representation of pH-responsive DOX/DQA-DOX@DSPE-hyd-PEG-AA for intracellular doxorubicin delivery in breast cancer. **(B)** Cytotoxicity of DOX, DOX/DQA-DOX@DSPE-hyd-PEG-AA and DOX@DSPE-hyd-PEG-AA in A549 cells at 48 h. **(C)** The distribution of DOX in mitochondria after A549 cells were treated with DOX@DSPE-hyd-PEG-AA **(a)** and DOX/DQA-DOX@DSPE-hyd-PEG-AA **(b)** at 37°C for 4 h. **(D)** *In vivo* antitumor activity of DOX/DQA-DOX@DSPE-hyd-PEG-AA in athymic nude mice xenografted with MDA-MB-231/ADR cells. Images were adapted with permission from reference (Song et al., 2015). Data are presented as mean ± SD, *n* = 4; ^∗^*p* < 0.05 and ^∗∗^*p* < 0.01, vs. normal saline; ^##^*p* < 0.01, vs. free DOX.

Song et al. (2015) further described DQA-Dox conjugate-loaded pH-responsive micelles to achieve drug resistance in cancer cells. The authors synthesized DQA-Dox conjugates via an amide linkage and DSPE-hyd-PEG-anisamide (AA) conjugates by the reaction between DSPE (1,2-distearoyl-sn-glycero-3-phosphoethanolamine) and PEG via linkage with the hydrazone bond, with cleavability at a low pH, with the aim of disassembly in lysosomes/endosomes (**Figure 8A**). To the DSPE-hyd-PEG conjugates was attached AA for active targeting to sigma receptors expressed on cancer cells. The Dox/DQA-Dox was loaded into micelles and assessed in drug-resistant cancer cells. A cytotoxicity study in MDA-MB-231/ADR cells revealed that the Dox/DQA-Dox-loaded nanoparticles had much higher activity (**Figure 8B**). A subcellular localization study using confocal microscopy showed that the nanoparticles were preferentially localized in mitochondria (**Figure 8C**). Furthermore, an antitumor study illustrated that nanoparticles with 2 and 10 μmol/kg of Dox had 2- and 5-fold greater tumor suppression effects than Dox (10 μmol/kg) in a Dox-resistant tumor model (**Figure 8D**). Mallick et al. (2015) directly conjugated α-TOS, a mitochondria-targeting drug, to cisplatin, Dox, or paclitaxel via monocarboxylate, amide, or ester bonds, respectively. The conjugates were then loaded into DSPE-PEG micelles with a size of less than 200 nm. The conjugate-loaded NPs showed much higher cell-killing effects than the free drugs, while their efficacy decreased after loading the conjugates in NPs. These studies revealed that conjugate-loaded nanoparticles localized in mitochondria and damaged the mitochondrial membrane, leading to cytochrome c release and cell apoptosis. The α-TOS-paclitaxel conjugate damaged the tubulin of HeLa cells.

## Conclusion

To date, many studies have investigated mitochondria-targeted drug delivery, particularly anticancer drug delivery. Early studies were focused on the direct conjugation of the mitochondria-targeting ligand and anticancer agents. Although direct conjugates were able to solve some issues such as drug-resistance, other challenges such as solubility problems remained, limiting preclinical and clinical applications. In the last decade, many studies have explored mitochondria-targeting carrier development using biomaterials and inorganic nanoparticles to solve the solubility and selectivity issues related to the drugs. Commonly applied mitochondria-targeting ligands are DLCs, particularly TPP and DQA, but those ligands are known to have toxicity problems. Therefore, some recent studies have described the replacement of TPP with other molecules, including short peptides and anionic molecules. Both direct conjugation and nanocarriers are commonly tested in drug-resistant cancer cells because drug resistance is one of the leading limitations of current chemotherapy, and in most cases, the conjugate and nanocarriers provide better results in drug-sensitive cells and animal models. In the future, more detailed preclinical and clinical studies with nanocarrier systems and the development of alternative targeting agents would become the core of the mitochondria-targeting drug delivery field. In addition, we noted that the most frequently used drug for both direct conjugation and nanocarrier for mitochondrial delivery was Dox. As there are several other mitochondria-mediated drugs, those drugs should also be studied.

## Author Contributions

GB, Y-YC, JL, HL, and HK collected context information and discussion. GB and HK wrote the manuscript.

## Conflict of Interest Statement

The authors declare that the research was conducted in the absence of any commercial or financial relationships that could be construed as a potential conflict of interest.
